# IMA Genome - F15: Draft genome assembly of *Fusarium pilosicola*, *Meredithiella fracta*, *Niebla homalea*, *Pyrenophora teres* hybrid WAC10721, and *Teratosphaeria viscida*

**DOI:** 10.1186/s43008-021-00077-9

**Published:** 2021-10-13

**Authors:** Tuan Anh Duong, Janneke Aylward, Claudio Gennaro Ametrano, Barsha Poudel, Quentin Carlo Santana, Pieter Markus Wilken, Anke Martin, Kiruba Shankari Arun-Chinnappa, Lieschen de Vos, Isabel DiStefano, Felix Grewe, Sabine Huhndorf, Helge Thorsten Lumbsch, Jostina Raesetsa Rakoma, Barsha Poudel, Emma Theodora Steenkamp, Yukun Sun, Magriet A. van der Nest, Michael John Wingfield, Neriman Yilmaz, Brenda Diana Wingfield

**Affiliations:** 1 Department of Biochemistry, Genetics and Microbiology, Forestry and Agricultural Biotechnology Institute (FABI), University of Pretoria, Private Bag X20, Hatfield, 0028 South Africa; 2 Department of Conservation Ecology and Entomology, Stellenbosch University, Private Bag X1, Matieland, 7602 South Africa; 3 Field Museum, Department of Science and Education, Grainger Bioinformatics Center, 1400 S Lake Shore Drive, Chicago, IL 60605 USA; 4 Centre for Crop Health, University of Southern Queensland, Toowoomba, QLD 4350 Australia; 5 Biotechnology Platform, Agricultural Research Council, Onderstepoort, Pretoria, 0110 South Africa; 6 PerkinElmer Pty LTD., Level 2, Building 5, Brandon Business Park 530-540, Springvale Road, Glen Waverley, VIC 3150 Australia

## IMA GENOME-F 15A

### Draft genome assembly of *Fusarium pilosicola*

#### Introduction

The *Fusarium fujikuroi* species complex (FFSC) is a diverse group of fungi with diverse ecologies that range from inhabiting soil to causing disease on a variety of plants (Kvas et al. 2009; Yilmaz et al. 2021). Members of this genus are also known for producing mycotoxins that are harmful to both human and animal health (Summerell 2019). Due to their economic importance, over 119 genome assemblies for species in the FFSC have been submitted to publicly available databases (https://ncbi.nlm.nih.gov), with the majority belonging to plant pathogens.

*Fusarium pilosicola* is a recently described FFSC taxon (O'Donnell et al. 2000; Yilmaz et al. 2021). The species was originally isolated in the USA from *Bidens pilosa*, commonly known as black-jack or cobblers pegs. Within the FFSC, *F. pilosicola* represents a sister clade to the economically important *Pinus* pathogen, *Fusarium circinatum* (O'Donnell et al. 2000; Yilmaz et al. 2021). *Fusarium pilosicola* shares morphological characteristics with *F. circinatum* and the closely related maize pathogen, *F. subglutinans*, but differ from them by being unable to produce the typical purple pigment in culture (Yilmaz et al. 2021). Other than the traits included in typical descriptions of *Fusarium* species, not much is known about *F. pilosicola*. The genome sequence of this species therefore provides an important resource to investigate its genetics, overall biology and evolutionary history.

#### Sequenced strain

**USA**: *Florida*: Fort Pierce, 27.4467° N; 80.3256° W, isolated from *Bidens pilosa* (NRRL 29124, CMWF 1183, PREM 63216-dried culture) (Yilmaz et al. 2021).

#### Nucleotide sequence accession number

This Whole Genome Shotgun project has been deposited at DDBJ/ENA/GenBank under the accession JAGQDI000000000. The version described in this paper is version JAGQDI010000000.

#### Materials and methods

**Fig. 1:**
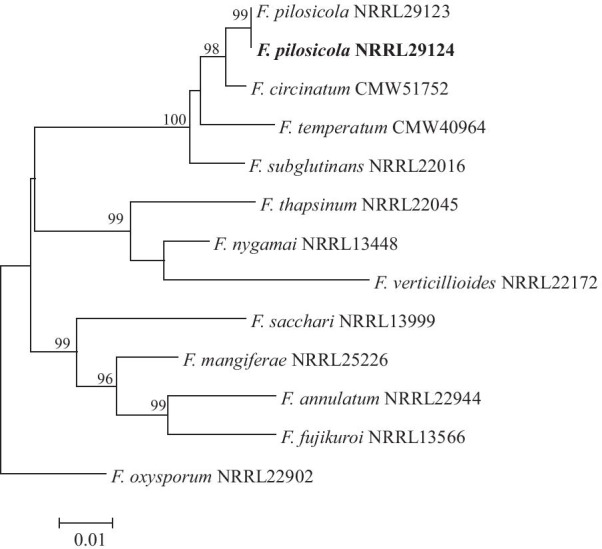
Maximum likelihood tree based on the partial gene sequences of translation elongation factor 1-α and β-tubulin (Herron et al. 2015; Wingfield et al. 2015, 2018). Sequence alignments were assembled with MAFFT v 7.472 (Katoh et al. 2019). The program jModelTest v 2.1.10 (Darribo et al. 2012) was used to determine the best-fit substitution model (TIM2 + G substitution model) with gamma correction (Tavare 1986). A maximum likelihood (ML) phylogenetic analysis was performed using PhyML v 3.1 (Guindon et al. 2010). Values at branch nodes are the bootstrapping confidence values with those ≥ 85% shown. The *F. pilosicola* isolate sequenced in this study was indicated in bold

*Fusarium pilosicola *was grown on half strength potato dextrose agar (BD Difco™) at 25 °C for 7 days after which genomic DNA was extracted as previously described (Möller et al. 1992). The DNA was then subjected to sequencing on the MinION sequencer (Oxford Nanopore Technologies) using a MinION flow cell (R10.3). The raw MinION data (coverage 178) was assembled into scaffolds using the Flye assembler (version 2.8.1) (Kolmogorov et al. 2019). The draft assembly was then subjected to two sequential rounds of error correction using RACON (version 1.4.17) (Vaser et al. 2017a, b). To confirm the identity of the sequenced isolate, we extracted relevant portions of the genes encoding translation elongation factor 1-α and β-tubulin, and used these in phylogenetic analyses (see the legend of Fig. 1 for detail).

Completeness of the genome assembly was evaluated with BUSCO (version 4.1.0) using Hypocreales_odb10 gene dataset (Seppey et al. 2019). Genome annotation was done with the MAKER pipeline (version 2.31.8) (Holt and Yandell 2011), which incorporated gene prediction tools AUGUSTUS (version 3.2.3) (Keller et al. 2011), GeneMark-ES (Ter-Hovhannisyan et al. 2008) and SNAP (Korf 2004). Gene model data from *F. circinatum* (Wingfield et al. 2012), *F. fujikuroi* (Wiemann et al. 2013), *F. verticillioides* and *F. graminearum* (Ma et al. 2010), as well as *F. mangiferae* and *F. proliferatum* (Niehaus et al. 2017), were included as additional evidence.

#### Results and discussion

The genome sequence of *F. pilosicola* (NRRL 29124) consisted of 95 scaffolds with a total genome size of 47 881 020 bp, an N50 value of 3 565 285 bp and G + C content of 47.04%. Phylogenetic analysis based on sequences extracted from this assembly, confirmed the taxonomic identity of the sequenced strain as *F. pilosicola* (Fig. 1). BUSCO analysis showed that the assembly was 97.5% complete, containing 97.3% complete and single-copy BUSCOs, 0.2% complete and duplicated BUSCOs; 2.2% fragmented BUSCOs, 0.3% missing BUSCOs (n = 4494). A total of 14 928 gene models were predicted using the MAKER annotation pipeline.

Sequence comparisons to the genomes of *F. circinatum* (NCBI accession: GCA_000497325.3) (Wingfield et al. 2018) and *F. temperatum* (NCBI accession: GCA_001513835.1) (Wingfield et al. 2015) indicated that scaffolds corresponding to all twelve of the known chromosomes for species in the FFSC are present in the *F. pilosicola* assembly. These comparisons also showed that the overall size and coding content of the *F. pilosicola* genome corresponded with those reported for other members of the so-called American Clade of the FFSC (O'Donnell et al. 1998). At 47.95 Mbp, the *F. pilosicola* genome is 2.45 Mbp larger in size than the 45.5 Mbp *F. temperatum* genome and 4.02 Mbp larger than the 43.93 Mbp *F. circinatum* genome. Despite these size differences, however, approximately 600 fewer genes were predicted for the *F. pilosicola* genome compared to those for *F. temperatum* and *F. circinatum*.

*Fusarium pilosicola* was isolated from *B. pilosa* in Florida which (O'Donnell et al. 2000) and its closest known relative is *F. circinatum* (Yilmaz et al. 2021), which is believed to have originated in Mexico and Central America (O'Donnell et al. 1998; Wikler and Gordon 2000). It was hypothesised that *F. pilosicola* moved with its noxious weed host when it expanded its range from the neotropics. Such an overlap in the geographic distribution these fungi’s plant hosts suggest their centres of origin likely also overlap, which would be consistent with their recent shared ancestry (Yilmaz et al. 2021). Availability of the genome of *F. pilosicola* will contribute to investigations into this phylogenetic relatedness.

*Authors*: Quentin C. Santana^*^, Lieschen De Vos, Neriman Yilmaz, Tuan A. Duong, Magriet A. van der Nest, Emma T. Steenkamp, Brenda D. Wingfield

*Contact: quentin.santana@fabi.up.ac.za

## IMA GENOME-F 15B

### Complete genome assembly of *Meredithiella fracta*

#### Introduction

Members of the fungal family *Ceratocystidaceae* have a well-established association with insects. For many genera this relationship lacks specificity (De Beer et al. 2014; Wingfield et al. 2013; Kirisits 2004), although some species form specific, mutualistic associations with ambrosial beetles (Mayers et al. 2020, 2015, 2018; Harrington et al. 2014). These fungi are carried in relatively large, specialized body cavities known as mycangia (Six 2003; Batra 1963). Adult beetles will inoculate the fungal symbiont from their mycangia into the sapwood of dead or dying trees where both the larvae and adult beetles will feed on the fungal growth (Harrington 2005). *Ceratocystidaceae* species which are ambrosial symbionts were historically grouped in the genus *Ambrosiella* (Harrington et al. 2010), although this group was plagued by a lack of monophyly (Massoumi Alamouti et al. 2009; Mayers et al. 2018). Subsequent studies have recognized four bark-beetle associated genera additional to *Ambrosiella*. These are the genera *Phialophoropsis* associated with beetles from the tribe *Xyloterini* (Mayers et al. 2015), *Toshionella* as symbionts of Asian *Scolytoplatypus* beetles (Mayers et al. 2020), *Wolfgangiella* as ambrosial fungi of African *Scolytoplatypus* species (Mayers et al. 2020) and *Meredithiella* for symbionts of the beetle genus *Corthylus* (Mayers et al. 2018).

Currently three species are recognized in the genus *Meredithiella*, although some cryptic taxa remain unresolved (Mayers et al. 2015, 2018). *Meredithiella norrisii* is the type species of the genus and was first described from galleries of *Corthylus punctatissimus* in the USA (Mayers et al. 2015). Subsequently, this fungus was also found in *C. columbianus*, also from the USA (Mayers et al. 2018). *Meredithiella guianensis* was isolated from the galleries and mycangia of *Corthylus crassus* from French Guiana, and is morphologically very similar to *M. norrisii* with size differences in the aleurioconidia being a distinguishing characteristic (Mayers et al. 2018). The third species, *M. fracta*, was isolated from the mycangium of *Corthylus papulans* beetles from the USA and Honduras (Mayers et al. 2018).

In this study a chromosome-level assembly of the ex-holotype of *M. fracta* is presented. This genome was produced using long-read nanopore sequencing together with short-read Illumina sequences. This genome is the first for any species in the genera *Phialophoropsis*, *Toshionella*, *Wolfgangiella*, or *Meredithiella*, and complements the two *Ambrosiella* genomes currently publicly available (Vanderpool et al. 2017; Wilken et al. 2020).

#### Sequenced strain

**USA**: *Florida*: Alachua County, Gainesville, dried culture isolated from mycangium of *Corthylus papulans,* 23 Feb 2016, *C. Bateman* (BPI 910531—holotype, dried culture; CBS 142645—ex-holotype culture).

#### Nucleotide sequence accession number

This Whole Genome Shotgun project has been deposited at DDBJ/ENA/GenBank under the accession JAGXCV000000000. The version described in this paper is version JAGXCV010000000.

#### Materials and methods

The ex-holotype isolate of *Meredithiella fracta* (CBS142645) was obtained from the culture collection of the Westerdijk Fungal Biodiversity Institute, Utrecht, The Netherlands. The culture was grown on YM (0.5% yeast extract, 2% malt extract, Biolab, South Africa) at 25 °C for 5 days. The QIAGEN Genomic-tips (Qiagen, Germany) was used to extract DNA from the culture using the protocol for plants and filamentous fungi, which was used to generate Nanopore sequencing reads on the MinION sequencing device (Oxford Nanopore Technologies, Inc., Oxford, UK). A sequencing library was prepared using the Genomic DNA by Ligation kit (SQK-LSK109) and was loaded on a MinION flow cell (R10.3) for a 48 h sequencing run. Base calling was conducted using the ONT Guppy basecalling software v 4.0.14. The resulting Nanopore reads were loaded onto the Galaxy bioinformatics platform (https://usegalaxy.eu); (Afgan et al. 2016), and used to generate a draft genome assembly with the Canu v 2.1.1 assembler (Koren et al. 2017).

Illumina sequencing data were generated at the Agricultural Research Council Biotechnology Platform (ARC-BTP; Pretoria, South Africa). The *M. fracta* DNA was used to prepare a pair-end library with a median insert size of 550 bp. An Illumina HiSeq 2500 instrument (Illumina, San Diego, USA) was used to generate paired-end reads of 150 bp. The raw reads were uploaded to Galaxy (Afgan et al. 2016) as separate libraries. Any adaptor sequences and low-quality reads were trimmed from the read libraries using a combination of Trimmomatic v. 0.38.1 (Bolger et al. 2014), Cutadapt (Martin 2011) and Trim Galore! v. 0.4.3.1 (a wrapper of the Cutadapt program). The trimmed illumina paired-end data were mapped to the Nanopore genome assembly using BWA-MEM (Li and Durbin 2009), and the resulting BAM file was used to polish the Nanopore assembly using Pilon v. 1.20 (Walker et al. 2014a, b).

The number of protein coding genes present in the genome was estimated in Galaxy using the AUGUSTUS de novo prediction software v. 3.3.3 with *Fusarium graminearum* gene models (Keller et al. 2011; Stanke et al. 2006a, b). Genome statistics including genome length, GC content, N50 and L50 values were calculated using QUAST v. 5.0.2 (Mikheenko et al. 2018), while completeness against the Fungi_odb10 and Ascomycota_odb10 datasets were determined using the Benchmarking Universal Single Copy Orthologs tool (BUSCO v. 5.0.0) (Simão et al. 2015) implemented using default parameters. To confirm the identity of the sequenced strain, the eukaryotic translation elongation factor 1 alpha (TEF1) gene was extracted from the assembly of *M. fracta* using CLC Main Workbench v21.0.2 and used together with representative sequences from other Ceratocystidaceae species in a phylogenetic analysis. To do this, the one-click mode of the Phylogeny.fr online tool (Dereeper et al. 2010, 2008) that includes alignment via MUSCLE (Edgar 2004), Gblocks alignment refinement (Castresana 2000) and tree construction using PhyML (Guindon et al. 2010; Guindon and Gascuel 2003) was used. An approximate likelihood-ratio test was used to determine branch support (Anisimova and Gascuel 2006).

#### Results and discussion

**Fig. 2:**
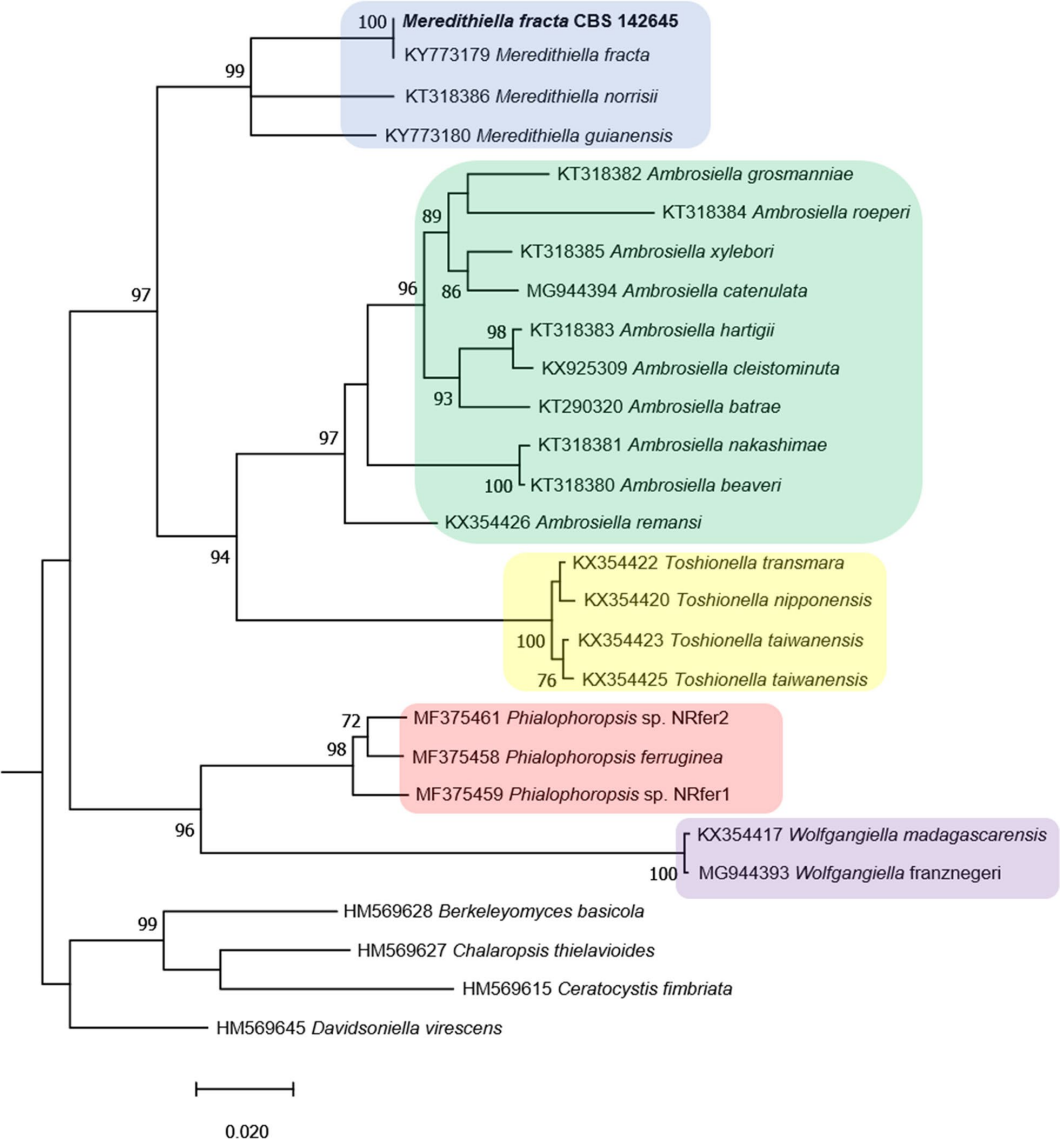
A maximum-likelihood phylogeny based on the *TEF1* gene from species of *Meredithiella* (blue), *Ambrosiella* (green), *Toshionella* (yellow), *Phialophoropsis* (red) and *Wolfgangiella* (purple). This analysis confirms the identity of the genome assembly presented here (shown in bold) as *Meredithiella fracta*. *Berkeleyomyces basicola*, *Chalaropsis thielavioides*, *Ceratocystis fimbriata* and *Davidsoniella virescens* were used as outgroups, and the results for the approximate likelihood ratio test for branch support are shown as percentages

The nuclear genome assembly of *M. fracta* had a length of 27,045,695 bp present in 11 scaffolds, of which 9 were 1.3 Mb or larger. The genome had a GC content of 46.64%, a N50 value of 3,970,047 bp and a L50 value of 3. The BUSCO analyses reported good completeness scores (89.2% for the 1706 ortholog Ascomycota dataset and 93.6% for the 758 ortholog Fungi dataset), with 1522 complete and 118 missing orthologs in the Ascomycota set and 710 complete and 26 missing orthologs in the Fungi dataset. AUGUSTUS de novo gene prediction using the *F. graminearum* gene models predicted 6296 protein coding genes, while phylogenetic analysis of the *TEF1* gene confirmed the identity of the isolate as *M. fracta* (Fig. 2).

The *M. fracta* genome has been assembled to a high level of completeness. Nine scaffolds are larger than 1 Mb, corresponding to the predicted number of chromosomes for the *Ceratocystis* species *C. fimbriata* and *C. manginecans* (Fourie et al. 2019). The two additional scaffolds that make up the *M. fracta* assembly are orders of magnitude smaller (~ 21 kb and ~ 31 kb), and likely represents unassembled fragments of the genome rather than accessory chromosomes. This is supported by the fact that none of the 6296 putative protein coding genes were predicted on either of these two chromosomes. An earlier comparative genomics study on *C. albifundus* also found that the genome contains no dispensable chromosomes, with accessory genomic components spread throughout the genome (Van der Nest et al. 2019).

*Meredithiella fracta* is the first of several genomes currently being sequenced for species of *Phialophoropsis*, *Toshionella*, *Wolfgangiella* and *Meredithiella* (Wilken et al. unpublished). These fungal lineages have only recently been delineated (Mayers et al. 2015, 2020), but have already impacted the study of ambrosial symbionts (Nel et al. 2020; Skelton et al. 2018; Vanderpool et al. 2017). The availability of full genome sequences for these and other Ceratocystidaceae ambrosial fungi (such as *Ambrosiella*; Vanderpool et al. 2017; Wilken et al. 2020) will add a genomic toolset for use in future studies. This includes genomes with a high level of accuracy (such as that of *A. cleistominuta* with a 98% BUSCO value; (Wilken et al. 2020) as well as assemblies with a high level of completeness (e.g. the genomes of *A*. *xylebori* (38 scaffolds; Vanderpool et al. 2017) and *M. fracta* (11 scaffolds; current study)). These could support a range of studies in future, including understanding the beetle-fungus symbiosis, comparative genomics of ambrosial fungi and developing tools for taxonomic studies on these fungal groups.

*Authors:* P. Markus Wilken*, Jostina R. Rakoma, Tuan A. Duong, Brenda D. Wingfield

*Contact: Markus.Wilken@fabi.up.ac.za

## IMA GENOME-F 15C

### Draft genome sequence of *Niebla homalea* (*Ramalinaceae*)

#### Introduction

*Ramalinaceae* is the fourth-largest family of lichenized ascomycetes with 42 genera and 913 currently accepted species exhibiting considerable morphological variation (Kistenich et al. 2018), and with 42 species of fruticose lichens which are recognized in the genus *Niebla* (Spjut 1996). As the genus name suggests, species of this genus thrive in fog-dependent ecosystems; the genus *Niebla* is indeed endemic to the New World coastal fog deserts (Sérusiaux et al. 2010). It occurs in two geographically disjunct coastal fog deserts along the Pacific coast of North and South America, the Baja California Peninsula and the Atacama Desert of northern Chile (Rundel 1978a). In this peculiar environment, the combined effects of frequent conditions of high atmospheric humidity and strong sea breezes restricts vascular plant vegetation and allows a remarkable lichen community to develop (Rundel et al. 1972) which express a relatively high levels of endemism at both the species and genus levels (Rundel 1978a, b; Rundel et al. 1991).

*Niebla homalea*, previously named *Desmazieria homalea* (Montagne 1852; Rundel 1978b), is the type species of the genus *Niebla*, and one of the most representative species of coastal deserts (Rundel et al. 1972). It occurs in association with other *Niebla* species and other species adapted to this environment (e.g. *Vermilacinia*, *Roccella*), which are able to efficiently exploit fog water by means of their growth form (Stanton and Horn 2013) and poikilohydric life-style (Green et al. 2011).

Within fruticose *Ramalinaceae* lichens, species are well resolved in the genera *Namibialina* and *Ramalina*. Also, the sister clade, *Vermilacinia,* is relatively well resolved, supporting the current species delimitation (Spjut et al. 2020). Species delimitation in *Niebla* is less straightforward; secondary metabolite variation generally coincides with major clades from molecular phylogenegetics, however, these clades often include samples from different morphologically described species (Spjut et al. 2020). A recent attempt to use next-generation sequencing, such as RADseq, provided crucial information about the diversification history of *Niebla*, however, it remained challenging to completely resolve this group (Jorna et al. in review).

We sequenced the first draft genome of the lichenized fungus *Niebla homalea.* The specimen for the mycobiont isolation was collected at the Shell Beach in the Sonoma Coast State Park, California. *Niebla homalea* is morphologically characterized by a fruticose thallus that is divided into many large branches and often with numerous smaller branches. The cortex is glossy, yellowish to olivaceous green, and often has black patches (Spjut 1996).

Our genome sequence of *N. homalea* will serve as a reference for future phylogenetic and comparative genomics studies involving members of *Ramalinaceae*. The genome sequence may aid in obtaining a better understanding of genomic factors that make the *Niebla* genus endemic to coastal fog deserts, an environment with limited supply of liquid water and characterized by harsh abiotic conditions. Furthermore, as the lichen communities of coastal deserts will face further challenges in the near future, such as those determined by climate change and the increase of land consumption in coastal areas, the genome sequence of *N. homalea* will also be of great value in a conservation genomics perspective.

#### Sequenced strain

**USA**: *California*: Sonoma Coast State Park; 38.420805, − 123.111199; Shell Beach, collected on a large rock, 25 Jul/2018, *S.M. Huhndorf and T.J. Widhelm* (F C0395311F, dried specimen). Lichen-fungus culture: *S.M. Huhndorf*  Field Museum culture lab strain fmnib47.

#### Nucleotide sequence accession number

The draft whole-genome sequence of the lichen-fungus *Niebla homalea* has been deposited at DDBJ/EMBL/Genbank under the accession number JAHGAU000000000. The version described in this paper is version JAHGAU010000000. Accession for SRA data: PRJNA707213.

#### Materials and methods

Axenic cultures were produced from lichen spores and grown on media until the individual cultures reached sufficient sizes for DNA extraction. A high-molecular weight (HMW) DNA extraction of the lichen-fungal culture was performed following the protocol published by Benjamin Schwessinger (10.17504/protocols.io.exmbfk6) with modifications, as for the lichen-fungus genome sequencing of *Physcia stellaris* (Wilken et al. 2020). About 0.6 g of dried fungal culture material was flash frozen with liquid nitrogen and ground with a ceramic mortar and pestle, then allowed to reach room temperature. The ground material was incubated with 500 µL lysis buffer and 20 µL proteinase K at 64 °C up to 4 h, then cooled on ice for 5 min. To the cool mixture 100 µL KAc 5 M was added and incubated for 5 min on ice, then centrifuged at max speed at 4 °C for 10 min. The supernatant was added to 500 µL phenol:chloroform:isoamyl alcohol (25:24:1) and centrifuged at max speed at 4 °C for 10 min. The supernatant was added to 500 µL isopropanol and cooled at − 80 °C for 1 h. The isolated HMW DNA was precipitated at max speed at 4 °C for 30 min, washed twice with 1 mL 70% ethanol, and eluted in 50 µL TE buffer.

Isolated HMW DNA was converted into Nanopore libraries with the NBD103 and 1D library kit SQK-LSK 109 (Oxford Nanopore Technologies Inc., UK). The libraries were sequenced on a SpotON R9.4.1 FLO-MIN106 flowcell for 48 h, using a GridIONx5 sequencer. The raw sequencing data was basecalled with guppy v. 3.0.3 (https://pypi.org/project/guppy3/3.0.3/), then adaptor trimmed with Porechops v. 0.2.3 (https://github.com/rrwick/Porechop). In addition, the same DNA sample was converted into Illumina sequencing libraries with the Hyper Library construction kit from Kapa Biosystems (Roche GmbH, Germany) and paired-end sequenced for 251 cycles on a MiSeq Illumina sequencer using the MiSeq 600-cycle sequencing kit v. 3 (Illumina Inc., Ca, US). All raw Illumina reads were trimmed with Trimmomatic v. 0.33 (Bolger et al. 2014), setting a quality threshold of 10 (LEADING:10 TRAILING:10). Library construction and sequencing were done at the DNA services facility at the University of Illinois at Urbana-Champaign.

The Nanopore reads were assembled into contigs using Flye v. 2.8-b1674 (Kolmogorov et al. 2019, b) or Canu (Koren et al. 2017). These assembled contigs were further aligned twice with Nanopore raw reads for error correction and scaffolding by using bwa v. 0.7.17-r1188 (Li and Durbin 2010) within the Racon v. 1.4.13 pipeline (Vaser et al. 2017a, b). The resulting consensus contigs were further polished twice with the trimmed Illumina reads by using bwa within the Pilon v. 1.23 pipeline (Walker et al. 2014a, b). The assembly quality was benchmarked using QUAST v. 5.0.2 (Gurevich et al. 2013). The genome completeness was estimated by BUSCO 4.0.6 (Simão et al. 2015) using the ortholog data set for Ascomycota (1706 genes).

The polished genome assembly was pre-annotated with MAKER 3.01.03 (Cantarel et al. 2008) using *Aspergillus nidulans* as the gene model species in AUGUSTUS v. 3.4.0 (Stanke et al. 2006a, b). The identified genes were then functionally annotated by using (1) InterProScan v. 5.47-82.0 (Jones et al. 2014), (2) UniProtKB Swiss-Prot, and (3) TrEMBL databases (March 2020). The *Niebla* genes were also searched for homologs against the prior annotation of the lichen-fungus *Physcia stellaris* (Wilken et al. 2020) using tblastn (BLAST v. 2.2.31) with an E-value cutoff of 1e−30, which served as a fourth dataset for gene functionality. For the final functional annotation best gene annotations were manually selecting from these four datasets. Secondary metabolites were predicted using antiSMASH v. 6.0.0alpha1-820a4b7 (Blin et al. 2019).

The *N. homalea* genome identity was determined with phylogenetic analyses of the internal transcribed spacer (ITS) barcoding marker sequence, together with other five loci commonly used in fungal phylogenies: ribosomal large subununit (LSU), glyceraldehyde-3-phosphate dehydrogenase (GDP), RNA polymerase II largest and second largest subunits (RPB1, RPB2) and translation elongation factor 1-alpha (tefa). These loci were identified with a BLASTn search using *Niebla* sequences available on NCBI as queries. The ITS region (ITS1, 5.8S, and ITS2) and the other loci extracted from the *Niebla* assembly were aligned with 101 samples from 27 *Niebla* species (Spjut et al. 2020) using MAFFT v. 7.475 (Katoh and Standley 2013). The resulting alignments were manually curated to remove ambiguously aligned nucleotide positions. Bayesian phylogenetic analysis was conducted running BEAST v. 2.6.1 (Bouckaert et al., 2014). Tree topology was linked across the six loci while clock and site model were left unlinked. The substitution model was set to GTR + G for each locus partition with gamma shape estimated and five rate categories; substitution rates were estimated. Clock model and priors were set to default values. Sequences from *Vermilacinia procera* and *Ramalina farinacea* were added to the dataset as outgroup but without setting them as an outgroup prior. The Maximum Clade Credibility (MCC) tree was generated from 45,000 trees sampled from two BEAST runs of 5*10^8^ generations after 10% of the sampled trees was discarded as burn-in; runs convergence was inspected in Tracer v. 1.7 (Rambaut et al. 2018), effective sample size values were greater than 200 for each of the sampled parameters. The node support of the resulting phylogenetic tree was evaluated as posterior probabilities in FigTree v1.4.2 (http://tree.bio.ed.ac.uk/software/figtree).

#### Results and discussion

The genome of the lichen-fungal culture *Niebla homalea* using Fly assembled into 52 contigs with a total length of 50.6 Mb. The final assembly contained only contigs larger than 25 Kb, with the largest being 3.158 Mb (Table 1). This assembly was used for further analyses since it outperformed the Canu assembly (111 contigs, N50 = 973 Kb). All contigs resulted in a N50 of 1.266 Mb with a GC content of 37.96%. The mean assembly coverage was 22× of Nanopore sequences and 21× of Illumina sequences. BUSCO analysis estimated a genome completeness of 96%, with only 0.2% duplicated genes and 4% fragmented or missing genes out of the 1706 Ascomycota single copy orthologs searched. Augustus gene modeling predicted a total of 9355 genes, resulting in an average gene density of 185 genes/Mb. These genes were interrupted by 17,778 introns; hence each gene contained on average 1.9 introns.

**Table 1:** Genome overview

Assembly	Flye	Canu
Assembly size (Mb)	50.57	51.63
Contigs	52	111
Contig N50 (Kb)	1267	972
Mean Coverage by Nanopore (fold)	22	22
Mean Coverage by Illumina (fold)	21	21
G + C content (%)	37.96	37.65
BUSCO completeness	C:96.0%[S:95.8%,D:0.2%],F:0.5%,M:3.5%	
**Gene prediction**		
Predicted gene models	9355	
Average gene length (bp)	1586.97	
Gene density (genes per Mb)	184.99	
Predicted introns	17 778	
Introns per gene	1.9	
**Secondary metabolites**		
Total SM clusters	44	
Type I polyketide synthetases (PKSs)	30	
Type III PKSs	1	
Nonribosomal peptide synthetases (NRPSs)	7	
NRPS-like fragments	4	
Terpene clusters	5	

The genome contained 44 gene clusters associated to secondary metabolite biosynthesis. Among them were identified 30 Type I and a single Type III polyketides synthetases (PKSs), 7 non-ribosomal peptide synthetases (NRPSs), and 4 NRPS-like fragments. In addition, antiSMASH identified 5 terpene gene clusters.

The draft genome is larger than the average for the Lecanoromycetes, the class to which *N. homalea* belongs, draft genomes sequenced to date (37.1 Mb; Apr 2021). In particular the *N. homalea* assembly is noticeably larger than the two other lichens belonging to the Ramalinaceae family whose draft genomes are publicly available: *Ramalina peruviana* (26.2 Mb) and *Ramalina intermedia* (27 Mb) (Wang et al. 2018). Moreover, the *N. homalea* assembly differs from most of the other sequenced Lecanoromycetes genomes in its GC content (37.96%), which is lower than the average of the class (46%). The difference is even more striking when considering the closely related *Ramalina* genomes, which have an average GC content of 51%. However, these differences could have been magnified by the different sequencing methods used. All *Ramalina draft* genomes were assembled from Illumina libraries while *N. homalea* was assembled from both Illumina and Nanopore libraries, which are less afflicted by GC bias (Browne et al. 2020).

The *N. homalea* genome assembled into a high-quality draft genome. The contiguity of the genome is comparable to other hybrid assemblies of lichen-forming fungi assembled from a combination of short and long reads technologies, or recent genomes only assembled from high coverage of long reads, such as the *Letharia columbiana* draft genome (McKenzie et al. 2020). Similar results were only obtained by using extremely high coverage of Illumina short reads, such as in *Cladonia metacorallifera* (Park et al. 2014) or *Umbilicaria muehlenbergii* (Park et al. 2014) draft genomes. However, it is generally difficult to obtain chromosome level genome assemblies by only relying on short reads and whole genome shotgun sequencing, as highlighted by the lower contiguity of most *Lecanoromycetes* draft genomes assembled form short reads only (data not shown).

The lack of a class-level orthologs database for *Lecanoromycetes*, limited the BUSCO completeness analysis sensitivity, however the *N. homalea* assembly performed in line with expectation and showed a similar completeness to the best assemblies produced so far within the class. The maximum BUSCO completeness in *Lecanoromycetes* draft genomes to date is 97.8%, reached by the highly contiguous (7 scaffolds) *Umbilicaria muehlenbergii* genome, while most other the high-quality draft genomes were at 94–97% completeness (data not shown).

**Fig. 3:**
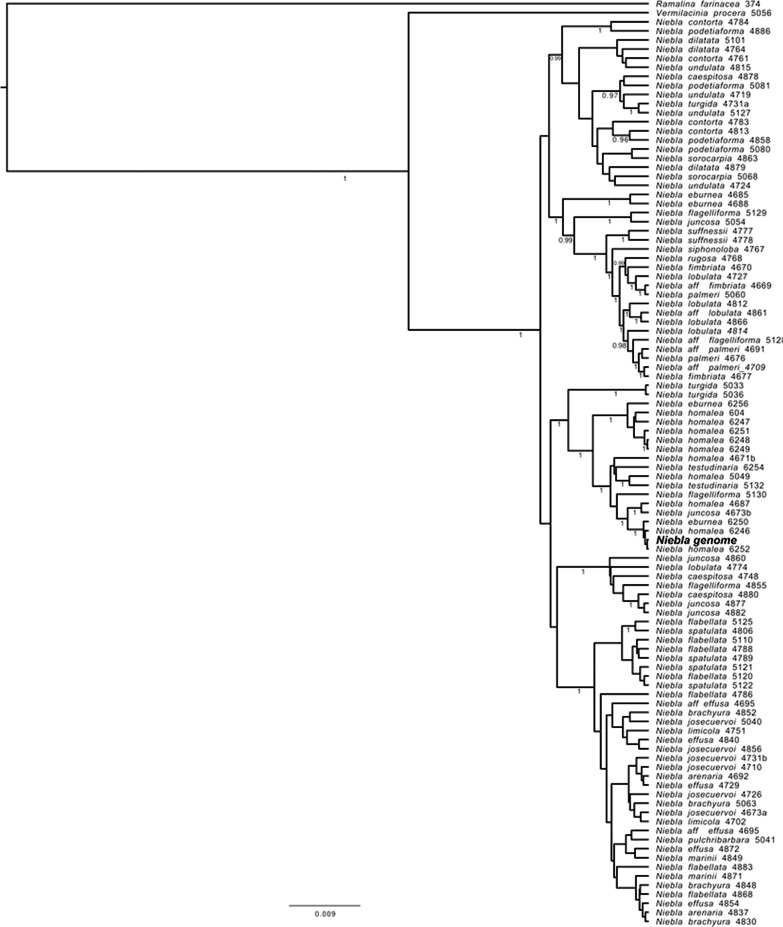
Bayesian six loci phylogeny including the genome sequence of *Niebla homalea* (highlighted in bold as ***Niebla* genome**). Posterior probability values above 0.95 are indicated below the branches. *Ramalina farinacea* and *Vermilacinia procera* were used as outgroup

The phylogenetic relationships and the species delimitation within the genus *Niebla* proved to be difficult to resolve following an integrative taxonomy approach, and even using multi-locus inferences and a wide taxon sampling it remains problematic (Spjut et al. 2020). Inference solely based on the ITS region, indeed, lacked the phylogenetic signal needed to confirm the species identity of the here assembled genome with confidence, as it was characterized by a lack of statistical support with many branches forming polytomies, especially using a maximum likelihood approach (data not shown). The six loci Bayesian phylogeny provided improved resolution (Fig. 3), allowing the identification of the sequenced draft genome as belonging to the clade of *N. homalea.* The clade where the genome sample was placed in our phylogeny is highly supported, and it is consistent with the one identified by the original phylogeny (Spjut et al. 2020), however, as mentioned, within this clade are present different morphologically described species.

The availability of the *N. homalea* draft genome from this study will allow comparative genomic studies within *Ramalinaceae*. It will also add to the genomic database of lichenized fungi for future research of evolutionary biology, aiming at an improvement of our understanding of molecular trends that were shaped by the lichen symbiosis. Moreover, *N. homalea* being endemic to coastal fog deserts, its genome sequence could be used for comparative analyses with cosmopolitan lichenized fungi able to colonize similar environments, in order to understand if there is any genome adaptation peculiar to coastal deserts lichens.

*Authors:* Claudio Ametrano* **Yukun Sun, Isabel DiStefano, Sabine Huhndorf, H. Thorsten Lumbsch, and Felix Grewe**

*Contact: Claudio Ametrano claudiog.ametrano@gmail.com

Table 1*Niebla homalea* draft genome overview. BUSCO completeness reported as percentage of complete (C), single copy (S), duplicated (D), fragmented (F), missing (M) genes. Basic statistics reported for both Flye and Canu assemblies. Flye assembly was used for all the subsequent analyses.

## IMA GENOME – F 15D

### Short-read genome assembly and annotation of a field collected *Pyrenophora teres* f. *teres* x *P. teres* hybrid WAC10721

#### Introduction

*Pyrenophora teres* f. *teres* and *P. teres* f. *maculata* are the causal agents of the net blotches, which are major foliar diseases of barley worldwide and may cause complete yield losses under favourable conditions (Liu et al. 2011; Mathre 1982; McLean et al. 2009). These haploid fungi occur as two forms, *P. teres* f. *teres* and *P. teres* f. *maculata*, based on the formation of net and spot like symptoms on barley leaves, respectively. Both forms co-exist in the field and produce sexual sporing bodies on stubble, releasing ascospores which act as the primary source of inoculum. Sexual reproduction between the two forms of *P. teres* has been induced in the laboratory and some of these *P. teres* hybrids have shown reduced sensitivity to triazole fungicides and were highly virulent on some barley genotypes (Campbell and Crous 2003; Campbell et al. 1999, 2002; Jalli 2011; Poudel et al. 2018). Hybrids between *P. teres* f. *teres* and *P. teres* exist in nature and to date two hybrids have been identified in Australia (McLean et al. 2014, 2020). One of the field hybrids collected in Western Australia was found to be rapidly spreading clonally and had resistance to some Group 3 compounds of azole or demethylation inhibitors fungicides (Lopez-Ruiz et al. 2020). The evolution of such new pathotypes would provide additional challenges when deploying resistant barley varieties. Therefore, to shed light on the genetic architecture and diversity of this emerging pathogen, we undertook the whole genome sequencing of Australian field collected hybrid, WAC10721. WAC10721 was characterised as a hybrid based on amplified fragment length polymorphism and *P. teres* form specific PCR markers (McLean et al. 2014; Poudel et al. 2017).

#### Sequenced strain

**Australia**: *Western Australia*: Esperance, isolated from *Hordeum vulgare*, Dec. 2002, *S. Gupta* WAC10721 (BRIP71571).

#### Nucleotide sequence accession number

This whole-genome shotgun project has been deposited in NCBI GenBank database under accession number JACXVK000000000 (BioProject: PRJNA656142 and BioSample: SAMN15768574). This paper describes the first version of this genome.

#### Materials and methods

Genomic DNA of WAC10721 was extracted from 10-day-old mycelium using a Wizard® Genomic DNA Purification kit (Promega, Sydney, Australia) as per the manufacturer’s protocol. The DNA was sent to Australian Genome Research Facility (AGRF), Melbourne, where integrity of DNA samples was confirmed by Agilent 2100 Bioanalyzer, agarose gel electrophoresis, and NanoDrop^TC^ 2000/2000c. The shotgun DNA library was constructed by AGRF as per Illumina’s protocol and sequenced with 125 bp pair-end reads on Illumina HiSeq 2000. The sequence quality of the pair-end reads were examined using FastQC (Galaxy v.0.72) (Afgan et al. 2018).

The adapter sequences were removed and reads with average phred score lower than 30 bp were filtered in the CLC Genomics Workbench v9.5.4 (hereafter referred to as CLC). De novo whole genome assembly was performed in CLC by adjusting word size from 20 to 40 bp using CLC default parameters (Arun-Chinnappa and McCurdy 2015; Henkel et al. 2012). Assembly quality was assessed using QUAST v5.0.2 (Gurevich et al. 2013). Repeat elements were detected and then masked using RepeatModeler v1.0.11 with Repbase v20.4 library (Bao et al. 2015) and RepeatMasker v4.0.9, respectively (http://www.repeatmasker.org).

For de novo gene annotation, the first round of MAKER2 v2.31.10 (Holt and Yandell 2011) was completed with AUGUSTUS v3.3.3 (Stanke et al. 2006a, b) using model organisms *Neurospora crassa* as training set and a self-trained GeneMark-ES v4.46 (Ter-Hovhannisyan et al. 2008) along with coding DNA and protein sequences of *P. teres* f. *teres* 0–1 (Ellwood et al. 2010) as gene and protein evidences. The gene models obtained from the first MAKER2 run were then used to train the ab initio annotation program SNAP (Korf 2004) and the second round of MAKER2 was rerun with a SNAP training output was used in the second round of Maker to further refine gene models. BUSCO v.4.1.2 (Simão et al. 2015) with fungi_odb10 database (758 core genes) was used to evaluate the completeness of the genome assembly. Interproscan v5.38-76.0 (Jones et al. 2014) was used to functionally annotate the predicted proteins and to assign gene ontology (GO) terms for the annotation data.

OrthoFinder v2.3.3 (Emms and Kelly 2015) was used to cluster the predicted proteins into orthologous groups together with *P. teres* f. *teres* and *P. teres* f. *maculata* proteomes (W1-1 and SG1) (Syme et al. 2018). SignalP v5.0 (Armenteros et al. 2019) and TMHMM v2.0 (Chen et al. 2003) were used to identify signal peptide and transmembrane domains in the predicted proteins. The proteins with the presence of a signal peptide and zero or one transmembrane domains were subjected to effector identification using EffectorP v2.0 (Sperschneider et al. 2018). The secondary metabolites were predicted using the assembly as input to the web-based antiSMASH 2.0 (Blin et al. 2017).

#### Results and discussion

**Fig. 4:**
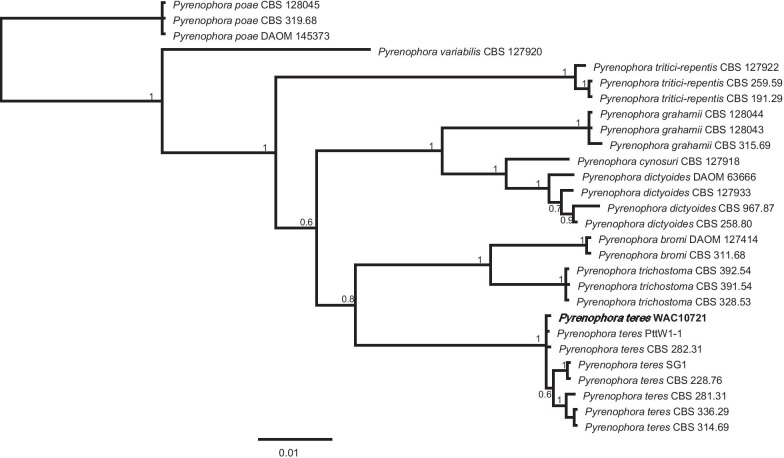
The phylogeny was constructed by Bayesian inference using MrBayes (Huelsenbeck and Ronquist 2001). The sequences of four loci ITS, LSU, *gapdh* and *tef1* were either obtained from the study of Marin-Felix et al. (2019), or extracted from the reference genome of *Pyrenophora teres* from Syme et al. (2018). Sequence alignments were produced using MAFFT v.7 (Katoh and Standley 2013)

Illumina sequencing generated 50 million reads on average accounting for ~ 100 × coverage of the whole genome. The genome was assembled into 2128 contigs that were equal to or larger than 500 bp. Total assembly length was 34.9 Mb with a GC content of 49.48% and a N_50_ value of 343,074. The longest contig consisted of 1,776,994 bp. We identified 1891 long terminal repeats, 695 DNA elements, 56 long interspersed nuclear elements, 4264 unclassified repeats, 7403 simple repeats and 750 low complexity regions. Repeats constituted of 10.54% of the genome. A total of 10,835 protein coding genes were predicted. BUSCO was used to assess the WAC10721 genome completeness and 98.6% of the core genes of fungi were identified. The basic statistics of assembly, repeat content and gene number for the reference genome of *P. teres* f. *teres* (W1-1), *P. teres* f. *maculata* (SG1) and the hybrid genome (WAC10721) are reported in Table 2. A phylogenetic tree reflecting the position of this genome in relation to other analysis of the genus *Pyrenophora* species is presented in Fig. 4.

**Table 2:** Genomic Statistics of the reference genome of *Pyrenophora teres* f. *teres* (W1-1), *P. teres* f. *maculata* (SG1) and the hybrid genome, WAC10721

Genomic statistics	Values		
	W1-1	SG1	WAC10721
Assembly size (Mbp)	51.76	41.28	34.90
Number of contigs (= > 500 bp)	74	47	2,128
N_50_ (bp)	3,700,000	2,110,000	343,074
GC-content (%)	45.21	46.86	49.48
Repeat Elements (bp)	3,115,060	1,061,852	3,419,914
Genes	11,245	11,165	10,835

The hybrid WAC10721 genome had 10,051 genes and these grouped in 9667 orthologous groups. In total, 9378 orthologous groups were common to all three isolates and WAC10721 shared 167 and 122 unique orthologous genes with the *P. teres* f. *teres* and *P. teres* f. *maculata* reference genome, respectively. Effector proteins and secondary metabolites play important roles in fungal pathogenicity and virulence (Moolhuijzen et al. 2020; Muria-Gonzalez et al. 2020; Wyatt et al. 2020). A total of 1039 predicted proteins in the genome have secretion signals and 18 of those were identified as effectors. In total 25 secondary metabolite gene clusters were identified comprising of 17 non-ribosomal peptide synthetases (NRPS), 14 polyketide synthases (PKS), three terpene and eight unclassified clusters. Files with information about the genome annotation, functional prediction, repeats, effectors and orthologous genes have been deposited on Zenodo with the DOI number (10.5281/zenodo.4107533). This is the first report of the genome of a *P. teres* hybrid. The data produced here will be useful for comparative genomics studies to understand differences between two forms of *P. teres* and hybrids and for investigating the mechanism of the pathogenicity of the species.

*Authors*: Barsha Poudel, Kiruba Shankari Arun-Chinnappa, Anke Martin^*^

*Contact: Anke.Martin@usq.edu.au

## IMA GENOME – F 15E

### A low coverage draft genome of the *Eucalyptus* leaf blight pathogen *Teratosphaeria viscida*

#### Introduction

*Teratosphaeria viscida *is one of a number of *Teratosphaeria* species that have emerged as pathogens of planted *Eucalyptus* trees over the past few decades (Burgess and Wingfield 2017, Andjic et al. 2019, Aylward et al. 2019). Unlike some other species, which are economically important globally, *T. viscida* is currently only known from *Eucalyptus* trials in tropical northern Queensland, Australia, where it has caused disease on *E. grandis* and *E. grandis* × *E. camaldulensis* trees (Andjic et al. 2007). Leaf blight caused by *T. viscida* was of particular concern when it was first identified because both the climatic conditions and host symptoms closely resembled those associated with disease outbreaks caused by *T. destructans*, one of the most devastating *Eucalyptus* foliar pathogens (Wingfield et al. 1996, Havenga et al. 2021).

On native *E. grandis* in Australia, *T. viscida* does not appear to be an aggressive pathogen, affecting < 15% of foliage and damaging only foliage in the lower tree canopy (Andjic et al. 2007). In contrast, an *E. grandis* × *E. camaldulensis* hybrid that was developed in South America and planted in Queensland became heavily infected within a year, with defoliation exceeding 95% (Andjic et al. 2007). These hybrid trees, stressed due to damage by *T. viscida*, were subsequently severely infected by *T. epicoccoides*, a species that typically causes only minor symptoms in healthy trees (Gardner and Hodges 1988, Taole et al. 2015, Andjic et al. 2019).

Phylogenetic studies have shown that *T. viscida* is closely related to other *Teratosphaeria* foliar pathogens that have asexual states in the genus *Kirramyces* (Andjic et al. 2019). These include *T. destructans*, a devastating pathogen across South East Asia and South Africa (Greyling et al. 2016, Havenga et al. 2021), and two other species, *T. novaehollandiae* and *T. tiwiana*, known only from Australia (Andjic et al. 2016). The aim of this study was to sequence the genome of *T. viscida*, as the closest relative of *T. destructans* with a genome available for comparative studies. Comparing the genome of *T. viscida* to those of aggressive pathogens such as *T. destructans* and *T. pseudoeucalypti* (Andjic et al. 2010) will also enable evaluation of the risk that this species poses as a potentially damaging pathogen of planted *Eucalyptus*.

#### Sequenced strain

**Australia**: *Queensland*: Mareeba, isolated from leaf spots on a *Eucalyptus grandis*, 2005, *T.I. Burgess* (CMW51324 = CBS121156 – culture).

#### Nucleotide accession number

The genomic sequence of *T. viscida* has been deposited at DDJ/EMBL/GenBank under the accession JAHESH000000000. This paper describes the first version.

#### Materials and methods

The culture of *T. viscida* is maintained in the culture collection (CMW) of the Forestry and Agricultural Biotechnology Institute (FABI), University of Pretoria, and the Westerdijk Fungal Biodiversity Institute (CBS), Utrecht, Netherlands. The isolate was grown on malt extract agar (Merck, Wadeville, South Africa) at 25 °C in the dark, until sufficient mycelia was available for DNA extraction, following methods previously described for *Teratosphaeria* species (Wingfield et al. 2018, Wilken et al. 2020). Extracted DNA was submitted to Inqaba Biotec (Pretoria, South Africa) for low coverage sequencing on the PacBio Sequel II System and to Macrogen (Seoul, Korea) for sequencing on the Illumina HiSeq 2500 platform. One Illumina paired-end library, with an insert size of 550 bp, was sequenced at a target read-length of 250 bp.

The quality of the raw Illumina sequence reads was assessed with FastQC 0.11.5 (Andrews 2010) and trimming was performed with Trimmomatic 0.38 (Bolger et al. 2014). Trimmed Illumina reads were assembled with SPAdes 3.14.0 (Bankevich et al. 2012), applying the “careful” option. Scaffolding was performed by aligning the PacBio reads to the SPAdes assembly with Minimap 2.17 (Li 2018) and running the Long Reads Scaffolder 1.1.11 (LRScaf; Qin et al. 2019). Both the Illumina and PacBio raw reads were used for final error correction of the hybrid assembly in Pilon 1.22 (Walker et al. 2014a, b). The completeness of the error-corrected assembly was assessed with BUSCO 4.1.4 using the Fungi *odb10* dataset (Simão et al. 2015). Repeat identification and gene prediction with the MAKER 2.31.10 pipeline (Holt and Yandell 2011) followed the method used for *T. gauchensis* and *T. zuluensis* (Wingfield et al. 2019). The *MAT1* locus of *T. viscida* was identified with BLASTn using the *MAT1* idiomorphs of *T. zuluensis* (GenBank accessions MN119556 and MN119557).

To confirm the taxonomic position of the *T. viscida* genome isolate, the Beta-tubulin, translation elongation factor-1 α (EF-1α) and internal transcribed spacer (ITS) regions were extracted from the genome. These were included in a maximum likelihood phylogeny with GenBank-obtained sequences from the ex-type isolates of other closely related *Teratosphaeria* leaf pathogens, using the two stem canker pathogens, *T. gauchensis* and *T. zuluensis*, as outgroup species. All sequences were aligned independently with MAFFT 7.407 (Katoh and Standley 2013), trimmed with trimAl 1.4.rev22 (Capella-Gutiérrez et al. 2009) and the best substitution model determined with ModelTest-NG 0.1.6 (Darriba et al. 2020) using the Akaike information criterion (AIC). Maximum likelihood (ML) phylogenies of the individual nucleotide alignments were constructed in RAxML-NG 1.0.2 (Kozlov et al. 2019), applying 1000 bootstrap replicates. A concatenated ML phylogeny was reconstructed after confirming congruence among the gene trees.

#### Results and discussion

Illumina sequencing yielded 1.6 million paired-end reads of which 68.9% were retained as paired and 13.3% as single reads after quality trimming. Mean length for the 38,050 PacBio reads was 3.8 kb, representing approximately 5X genome coverage. The final error-corrected hybrid genome assembly was 37.4 Mb, with an estimated coverage of 14X, a GC content of 49.1% and a BUSCO completeness score of 98.7%. It consisted of 1879 contigs with an N50 and L50 of 130 kb and 83 contigs, respectively. The size of the *T. viscida* genome was 6–11 Mb larger than that of other published *Teratosphaeria* genome assemblies (Wingfield et al. 2018, 2019; Havenga et al. 2020; Wilken et al. 2020), a characteristic reflected in its 28.7% repetitive fraction. Up to this point, the highest estimated repeat content for a *Teratosphaeria* pathogen had been approximately 17% for *T. destructans* (Wingfield et al. 2019; Wilken et al. 2020). The number of predicted genes (9528) for *T. viscida* was similar to that of other *Teratosphaeria* species.

**Fig. 5:**
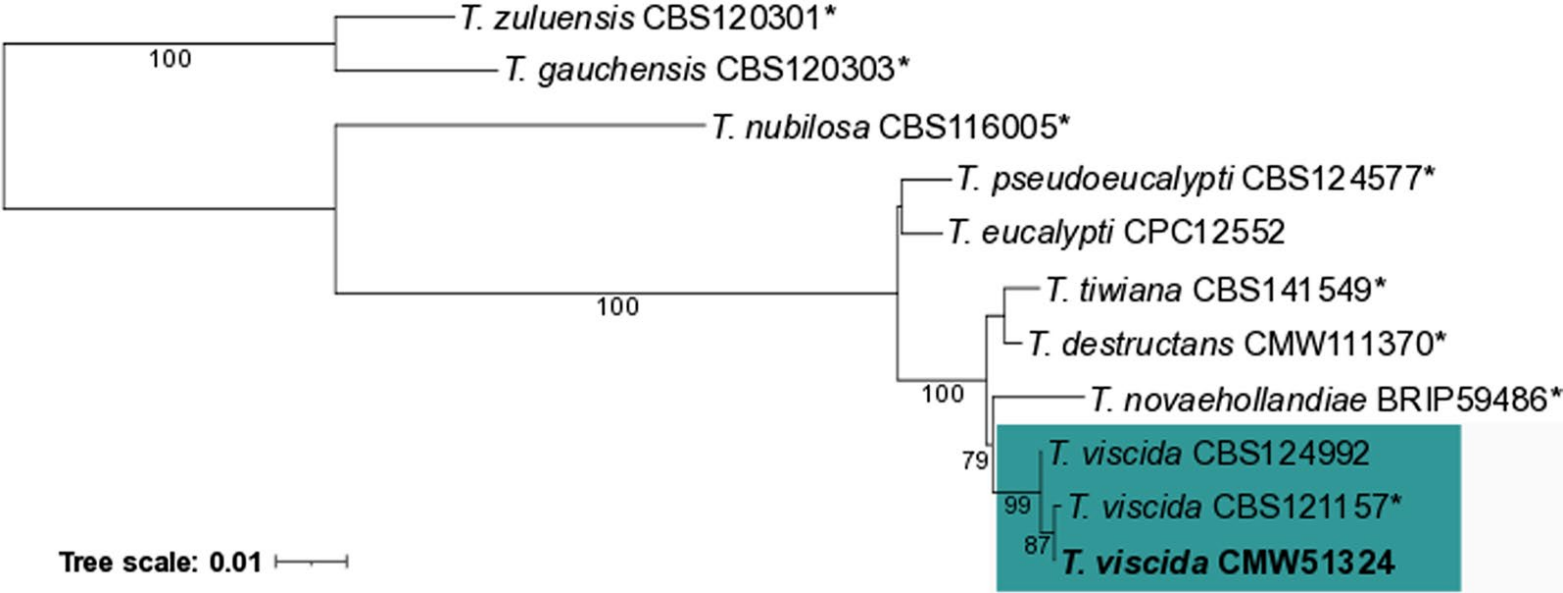
Maximum likelihood phylogeny of the concatenated Beta-tubulin, EF-1α and ITS gene regions indicating the taxonomic position of the *Teratosphaeria viscida* genome isolate (bold). Type isolates are indicated with an asterisk (*). Support values represent the transfer bootstrap expectation metric (TBE) of Lemoine et al. (2018). GenBank accession numbers are available in Quaedvlieg et al. (2014) and Andjic et al. (2016)

ML analysis of three concatenated barcoding genes confirmed that the genome strain resolves in a well-supported monophyletic clade with the ex-type strain of *T. viscida* (Fig. 5). It also confirmed the close relationship among *T. viscida* and the other species, *T. destructans*, *T. novaehollandiae* and *T. tiwiana*, having kirramyces-like conidia. BLASTn identified a *MAT1-1* idiomorph in the *T. viscida* genome, with the same gene content and flanking genes as that of *T. zuluensis* (Aylward et al. 2020). *Teratosphaeria viscida* is, therefore, heterothallic, similar to most other investigated *Teratosphaeria* species, the exception in this case being *T. nubilosa* (Hunter et al. 2009).

The genome sequence derived from *T. viscida* in this study is the first for a *Teratosphaeria* species that is apparently less aggressive than its closest relatives. Additionally, it is the only genome from a species known exclusively from eastern Australia. The genome of this species is a valuable addition to the dataset of *Teratosphaeria* genomes and will be a useful comparison with aggressive leaf pathogens for which genomes are already available, including *T. destructans*, *T. pseudoeucalypti* and *T. nubilosa* (Wingfield et al. 2018, Haridas et al. 2020, Wilken et al. 2020).

*Authors*: Janneke Aylward*, **Brenda D. Wingfield, Michael J. Wingfield**

*Contact: janneke.aylward@fabi.up.ac.za
